# Carbohydrate Reduction and a Holistic Model of Care in Diabetes Management: Insights from a Retrospective Multi-Year Audit in New Zealand

**DOI:** 10.3390/nu17243953

**Published:** 2025-12-17

**Authors:** Caryn Zinn, Jessica L. Campbell, Lily Fraser, Glen Davies, Marcus Hawkins, Olivia Currie, Jared Cannons, David Unwin, Catherine Crofts, Tom Stewart, Grant Schofield

**Affiliations:** 1 Human Potential Centre, School of Sport, Exercise & Health, Auckland University of Technology, Auckland 1142, New Zealand; 2 Turuki Healthcare—Mangere, Auckland 2022, New Zealand; 3 Reversal NZ, Taupo 3330, New Zealand; 4 Highbrook Medical Centre, Auckland 2013, New Zealand; 5 Real Healthy Me, Christchurch 8011, New Zealand; 6 Norwood Surgery, Southport PR9 7EG, UK

**Keywords:** carbohydrate reduction, prediabetes, remission, holistic, health-coaching

## Abstract

Background/Objectives: The global epidemic of type 2 diabetes (T2D) is a critical public health issue, particularly in New Zealand, where prevalence rates are high, especially among Māori and Pacific people. Recent research indicates that dietary interventions, particularly carbohydrate reduction, can lead to the remission or reversal of T2D. However, little is known about how such approaches perform when implemented in routine New Zealand primary care, particularly within high-risk and underserved populations. This study aimed to evaluate changes in HbA1c, diabetes status, and cardiometabolic outcomes among adults with prediabetes and T2D engaged in such a model of care. Methods: This study reports findings from a retrospective, observational, real-world, multi-site clinical audit (service evaluation) of a holistic model of care implemented in three primary care practices in New Zealand. The model of care is characterised by a three-pronged approach: whole food, carbohydrate reduction; a health-coach, behaviour-change-based delivery approach; and community- or peer-based initiatives. Audit data from 106 patients with prediabetes (PD) and T2D were analysed (median follow-up 19 months; IQR 6–32) to assess changes in glycosylated haemoglobin (HbA1c) levels, diabetes status, and cardiometabolic outcomes. Results: We observed an overall reduction in HbA1c (median change −3 mmol/mol (IQR: −7 to 3), *p* = 0.004), with 32% of patients with T2D at baseline achieving reversal and 44% of those with PD attaining normoglycaemia at final follow-up. Weight loss was associated with greater HbA1c reduction (0.56 mmol/mol decrease per kg lost) and additional improvements seen in lowered alanine aminotransferase (ALT). HDL cholesterol showed a small decline (r = 0.31), and triglycerides and blood pressure showed no significant change, indicating that these measures remained broadly stable over the evaluation period. Conclusions: Given the retrospective and uncontrolled audit design, findings should be interpreted with appropriate caution. However, the consistent improvements observed across multiple practices suggest that carbohydrate-reduction strategies within holistic models of care can meaningfully improve diabetes outcomes in real-world primary care settings. Future research should evaluate longer-term sustainability, implementation fidelity, and the applicability of this model at scale, particularly for Māori and Pacific communities.

## 1. Introduction

The global type 2 diabetes (T2D) epidemic is a significant public health crisis [1]. T2D is clinically diagnosed using established glycaemic thresholds, including an HbA1c ≥ 48 mmol/mol (6.5%), as recognised by major international guidelines [2]. Driven largely by unhealthy lifestyle factors such as energy-dense, nutrient-poor diets, excessive alcohol consumption, smoking, and physical inactivity, these behaviours contribute to insulin resistance, hyperinsulinemia, obesity, and ultimately T2D [3,4,5,6,7]. Importantly, many of these drivers are modifiable, highlighting the preventability of much T2D and the value of intervening early to improve metabolic health. In New Zealand, an estimated 6.4% of adults suffer from diabetes, and 20% suffer from prediabetes (PD) although these figures may underestimate the actual burden [8,9]. The burden is especially high among Māori, the Indigenous people of New Zealand, and among Pacific people living in New Zealand [10] and is projected to increase further over the next two decades [11]. These groups also face longstanding barriers to effective diabetes care, including cost, limited access to culturally aligned services, and structural inequities, which heightens the need for approaches that are community-based, acceptable, and tailored to local contexts [12]. Given the risk of related health issues including cardiovascular disease, cancer, and dementia [13,14,15,16], the impact on individuals, communities, the health system, and the economy is significant.

Historically, T2D has been considered a chronic, progressive condition; however, recent research shows that dietary interventions, particularly in early stages, can reduce insulin resistance, alleviating beta cell stress and improving insulin secretion [17]. This paradigm shift challenges the conventional view that T2D is irreversible, introducing terms such as ‘diabetes remission’ and ‘reversal’ into the T2D discourse. Since the introduction of dietary management strategies for T2D, such as very low-calorie approaches and carbohydrate reduction, these concepts of ‘diabetes remission’ and ‘reversal’ have gained prominence [17,18,19] and substantial evidence now supports the efficacy of carbohydrate reduction in managing and potentially reversing PD and T2D [20,21,22,23].

Beyond pharmacological interventions and bariatric surgery, T2D management and potential reversal have traditionally focused on weight loss [18]. Various dietary approaches—including very low-calorie, reduced-carbohydrate, low-fat, Mediterranean, and plant-based diets—have all demonstrated efficacy in both weight reduction and diabetes management [17,24,25,26,27,28,29,30]. Of these, reduced carbohydrate diets offer additional benefits for diabetic control, independent of weight loss [31,32]. While data on long-term adherence and safety were previously limited [33], recent studies have shown accumulating evidence supporting the safety and effectiveness of carbohydrate reduction for glycaemic control, medication reduction, weight loss, and reduction in cardiovascular risk factors [22,27,33,34,35,36,37,38] with some studies tracking these benefits over timeframes of up to five years [39,40,41]. Consequently, recent international consensus guidelines now endorse carbohydrate reduction as a legitimate therapeutic strategy for T2D [42,43,44].

While the efficacy of carbohydrate reduction is well established, less is known about its effectiveness, acceptability, and sustainability in routine clinical practice. Conventional T2D care predominantly involves one-on-one consultations between patients and healthcare providers, with primary emphasis on medication management using oral hypoglycaemic agents [45] and often excludes family involvement. Despite best practice guidelines advocating for lifestyle interventions, especially dietary guidance [46], primary care practitioners encounter significant constraints such as brief consultation periods and inadequate nutrition education, resulting in insufficient focus on dietary therapeutic approaches [47]. Although other health professionals, such as dietitians and pharmacists, could play key roles in primary care, they are not routinely integrated into the New Zealand primary care system [47]. With primary care practitioners facing escalating workloads and high attrition rates expected over the next decade [48] there is an urgent need for more comprehensive, integrated approaches to T2D management. The most significant workforce shift in the last couple of years has been the integration of health coaches into the primary care setting [49,50]. Health coaches typically provide personalised dietary support, behaviour-change guidance, and ongoing encouragement, helping patients implement and sustain lifestyle modification alongside clinical care. Such personnel are increasingly being recognised, alongside use of group sessions and support groups, as has been used successfully internationally [22,51,52,53,54].

Together, these factors highlight a critical gap: although dietary strategies such as carbohydrate reduction have an expanding evidence base, their implementation within routine New Zealand primary care remains limited, and no coordinated national strategy exists to guide their use. Understanding how such approaches perform in real-world clinical contexts, particularly for diverse and high-risk populations, is therefore an important and timely area of investigation. This study presents a retrospective, multi-site clinical audit of three primary care practices in New Zealand implementing a holistic three-pronged approach to holistic care: dietary change through whole-food, carbohydrate reduction, behavioural support delivered via a health- coach approach, and structured community initiatives providing ongoing support. The audit was designed as a service evaluation of existing clinical practice rather than a controlled trial, and results should therefore be interpreted as observational associations. An observational service-evaluation design is particularly suited to assessing implementation because it captures real-world variability, routine clinical workflows, and diverse patient presentations that are often excluded from traditional trials. The models of care were tailored to each clinic population but shared common features, including a focus on carbohydrate reduction, health coach–led support, digital tools for education and tracking, and community or peer-based initiatives to reinforce behaviour change. Using multi-year data from adults with PD and T2D, this audit aimed to (1) describe changes in glycosylated haemoglobin (HbA1c) and diabetes status, (2) identify factors associated with HbA1c improvement, and (3) examine changes in related cardiometabolic outcomes. Together, these findings provide insight into how holistic, diet-focused care can be implemented and evaluated in real-world primary care settings.

## 2. Materials and Methods

This study was a retrospective, observational, clinical audit conducted across three primary care practices in New Zealand. All three practices utilise a model of care aimed at managing and potentially reversing T2D and PD, using a three-pronged approach: (1) whole-food, carbohydrate-reduction, (2) a health-coach approach, and (3) supportive community initiatives. While the core components were consistent across practices—GP oversight, carbohydrate-reduction guidance, and access to health coaching—the way the model was applied (including session frequency, content, mode of delivery, and cultural tailoring) was left to the discretion of each practice to align with its individual context. Differences primarily related to funding models, community-based education opportunities, and the extent of group or peer-support initiatives available at each site, shaped by local demographics and cultural context.

In all practices, “health coaches” were non-physician practitioners with formal training in nutrition, lifestyle behaviour change, and motivational interviewing. All health coaches were formally trained through PREKURE [55], a highly professional, internationally accredited organisation whose Health Coach qualification is recognised by the Health Coaches Australia and New Zealand Association (HCANZA). Their role was to coach patients using dietary education, practical support, and ongoing behavioural techniques under GP supervision, consistent with their competencies.

### 2.1. Characteristics of the Health Care Centres

As described above, while the fundamental models of care are similar across practices, each tailors their approach to match patient demographics and available healthcare resources. Each practice is described below.

Practice 1: This is a publicly funded clinic where GPs and health coaches collaborate to optimise patient outcomes. Initially, the GP conducts a consultation, subsequently referring the patient to health coaches. Follow-up consultations with the GP focus on standard medical monitoring, including discussing existing conditions and ordering further blood work and medication adjustments.

Health coaches engage with patients regularly through a combination of face-to-face meetings and remote communications, such as texts, phone calls, and emails. These interactions emphasise education, counselling, practical application, motivation, and support for behaviour change. Patients benefit from weekly cooking classes and participation in the community initiative “Low Carb Healthy Fanau” (a play on “Fat” with “Fanau” meaning family in Samoan) via a dedicated Facebook page. Furthermore, a journaling app, developed by health coaches, features educational videos on carbohydrate reduction and lifestyle modifications to enhance patient support. A wide range of other programmes and services are available to patients covering aspects such as smoking cessation, healthy homes, and Māori meditation.

Practice 2: This is also a publicly funded clinic where patients receive standard 15 min consultations with a GP, who provides dietary advice on carbohydrate reduction. Following the initial consultation, patients may receive three months of health coaching and nutrition advice through a warm handover, whereby the clinician personally connects the patient with a local health coach to ensure continuity of care. Additional support is provided via a local Reverse T2 Diabetes initiative, which involves weekly 90 min meetings and a Facebook group for community support. These group meetings offer continuing education on diet, lifestyle changes, and associated physiology, with participation available both in person and online. Educational topics are regularly updated and cycled, with contributions from guest speakers. Health coaching at Practice 2 similarly focused on dietary support, behaviour-change guidance, and ongoing check-ins, though delivered with fewer structured group activities compared with Practice 1.

Practice 3: This is a specialised privately funded clinic focused on reversing PD and T2D, amongst other metabolic conditions. The clinic uses a flexible model, allowing patients to customise their care by adding services such as nutritionist consults, health coach follow-ups, or psychologist appointments after their initial GP visit. As per Practice 2, additional support beyond individual appointments is provided via the Reverse T2 Diabetes initiative. Health coaches at this practice work in a similar manner to those at Practice 2.

### 2.2. Patient Selection

During routine clinic appointments, patients with PD and T2D were identified by the GPs in an opportunistic manner. They were then offered the chance to manage their diabetes through carbohydrate-reduction and lifestyle modifications, and if they were interested, the referral was made to the health coach. No patients were excluded from care. Referral criteria were informal and based on clinician judgement during routine consultations rather than predefined eligibility thresholds.

Carbohydrate reduction generally involved lowering overall carbohydrate intake, aiming for below approximately 130 g per day [56], although specific targets were not systematically prescribed or tracked. Instead, reductions were guided by patient goals, clinical judgment, and ongoing discussions with health coaches or clinicians, as per protocol in general practice settings. A carbohydrate-reduced approach was implemented for patients using SGLT2 inhibitors, but a ketogenic diet was not prescribed for those taking empagliflozin (Jardiance) due to an increased euglycaemic diabetic ketoacidosis (DKA) risk; this precaution was routinely noted in GP referrals to health coaches. The intensity and duration of health-coach contact varied across practices and individuals; this was not formally standardised or evaluated, reflecting pragmatic real-world implementation.

### 2.3. Clinical Audit

Data for the clinical audit were collected for different durations across the three medical clinics. For Practice 1, data was collected between March 2013 and December 2023, for Practice 2 between June 2014 and August 2020, and for Practice 3 between January 2021 and May 2023.

Baseline data collected at all three practices included demographics (age, sex, duration of GP and health coach observations), glycosylated haemoglobin (HbA1c), and lipid panels including low-density lipoprotein cholesterol (LDL), high-density lipoprotein cholesterol (HDL), total cholesterol, and triglycerides. In addition, Practices 1 and 3 measured renal function, and liver function, while Practices 1 and 2 measured weight, and blood pressure. A summary table (Table 1) indicating which metabolic markers were measured at each practice is presented to support transparency regarding data availability.

Subsequent data were then collected during routine or necessary reviews with the same parameters as above measured for each practice. For the comparative analysis, the most recent health parameters of patients at the end of the observation period were utilised. This same point was also considered the endpoint for determining the duration of each patient’s observation.

While the broadly accepted definition of reversal is an HbA1c < 48 mmol/mol (6.5%) with or without metformin [20], here <50 mmol/mol (6.7%) was used, consistent with New Zealand guidelines [57]. This minor adjustment does not affect the clinical significance or timeframe of the definition.

At Practices 1 and 3, data were also collected on patient engagement. This included documenting whether patients were engaged with other initiatives (health coaching, cooking classes, community talks; measured as yes/no), the number of visits to health coaches during the initial period (n) and the frequency of GP visits (times per month). The ‘initial period’ referred to the more intensive early phase of care, during which patients typically had more regular contact with health coaches before transitioning to less frequent follow-up. The length of this period varied according to individual needs, reflecting standard practice in both clinics. Engagement metrics were not available at Practice 2, limiting direct comparison of engagement effects across all three sites.

No formal adverse-event monitoring framework was in place, although clinicians documented any clinically relevant concerns during routine follow-up, consistent with their usual practice.

### 2.4. Statistical Analysis

Data were analysed using R (version 4.2.3). Descriptive statistics, including median and interquartile ranges (IQR) for continuous variables, and percentages for categorical variables, were used to summarise patient demographics, clinical characteristics, and diabetes status across the three practices.

To address Aim 1, the number of patients who achieved reversal of T2D between their baseline and final follow-up measurements was described. Patients who only had a single measurement were excluded from these comparisons to ensure the reliability of the results. Transitions in diabetes status are presented using an alluvial chart. HbA1C values were compared across the three practices by specifying HbA1c as the dependant variable, while practice (Practice 1, 2, or 3), and time (baseline, final follow-up) were specified as fixed effects, along with their interaction, in a linear mixed-effect model. Follow-up duration (number of days) was added as an additional covariate. Patient was specified as the random effect to acknowledge the repeated measures.

To investigate which factors were associated with changes in HbA1c levels (Aim 2), a series of linear mixed-effect models were used. The change in HbA1c (mmol/mol) between baseline and final follow-up was specified as the dependant variable in all models. Model 1 included patients from all practices (n = 103) with baseline HbA1c (mmol/mol), age (years), follow-up duration (days), and ethnicity (European, Māori or Pacific, Other) as fixed effects. Model 2 (n = 75, patients from Practices 1 and 2) added weight change (kg), and Model 3 (n = 51, patients from Practices 1 and 3) included patient engagement metrics (yes/no), number of visits during the initial period (n) and frequency of doctor visits (times per month). Three separate models were run to maximise the number of observations, as key predictors (e.g., weight change, engagement metrics) were not consistently available across all practices. To account for the clustering of patients within medical centres, the medical organisation was specified as a random intercept. More complex random effect structures were explored (i.e., random slopes for key predictors) but these did not result in substantive improvements in model fit. The mixed models were fitted using the *lme4* package in R, and estimated marginal means for each fixed effect were computed using the *emmeans* package.

Changes in cardiometabolic outcomes (weight, blood pressure, lipid profile, liver, and renal function) between baseline and final follow-up were assessed using the Wilcoxon signed-rank test for paired data (Aim 3). The effect size (r) for the differences was calculated using the formula r = z/sqrt(N)) [58], where N is the number of matched pairs. Effect sizes of 0.10 to less than 0.3 indicates a small effect, 0.30 to less than 0.5 indicates a moderate effect, and 0.5 or greater indicates a large effect. Given the exploratory nature of the audit, the cardiometabolic outcomes were treated as separate dependant variables and no formal adjustment for multiple testing was applied; effect sizes are presented to aid interpretation.

### 2.5. Ethics

Clinical audits of service do not usually need to go through an ethics approval process as they are a routine part of quality assurance in the medical setting. However, in New Zealand, clinical audits that have been funded by the Health Research Council, including this work, must undergo ethics approval. Hence, this study was approved by AUTEC (AUT Ethics Committee) on 10 October 2022; reference number 22/241. As a retrospective audit of existing clinical records, patient informed consent was not required. Data management agreements were established between each practice and the University. All data were de-identified prior to analysis and stored securely on password-protected institutional servers in accordance with data-governance requirements.

## 3. Results

Across the three practices, 106 patients chose to manage their T2D or PD with a reduced carbohydrate approach. Data were not available for patients who declined or pursued alternative management strategies, so findings reflect those who engaged with the programme. Demographic characteristics (Table 2) varied markedly between practices, with Practice 1 having a predominantly young Māori and Pacific population, Practice 2 mostly European and older, and Practice 3 European and younger on average. The median follow-up duration for HbA1c across practices was 19 months (IQR 6–32).

### 3.1. Blood Sugar Control

Across the three practices, the median HbA1c levels showed a significant reduction from 51 mmol/mol (IQR: 42 to 65) at baseline to 47 mmol/mol (IQR: 41 to 59) at the final follow-up, with a median change of −3 mmol/mol (IQR: −7 to 3). A Wilcoxon signed-rank test confirmed that this decrease was statistically significant (z = 3412, *p* = 0.004, r = 0.28). A subgroup analysis of patients who recorded a reduction in HbA1c at follow-up (n = 64), showed a larger median improvement (z = 2080, *p* < 0.001, r = 0.87). These patients showed a median decrease from an initial HbA1c of 52 mmol/mol (IQR: 43 to 70) to 43 mmol/mol (IQR: 40 to 51).

**Figure 1:**
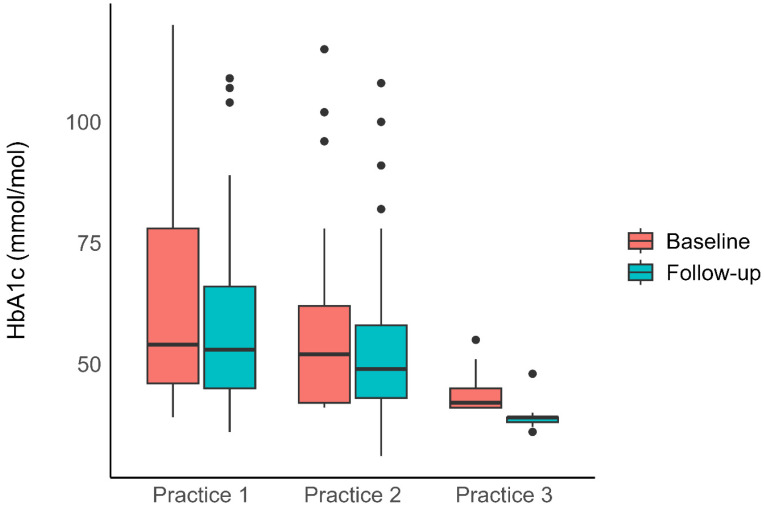
Box and whisker plot showing baseline and final HbA1c levels for each of the three practices.

Patients were split between the three practices with similar decreases observed at Practices 3 and 2; change of −5 (IQR: −6 to −3, n = 18) and change of −5 (IQR: −9 to −2, n = 22), respectively, while a larger change was observed at Practice 1; −10 (IQR: −24 to −6, n = 25). A linear mixed-effect model suggested that while there was a significant main-effect of practice, F(2, 186) = 24.7, *p* < 0.001, the time × practice interaction suggested the HbA1c decrease between timepoints was similar across the three practices F(2, 111) = 0.19, *p* = 0.82. Figure 1 presents a box-and-whisker plot illustrating the baseline and final HbA1c levels for each of the three practices.

### 3.2. Diabetes Reversal

**Figure 2:**
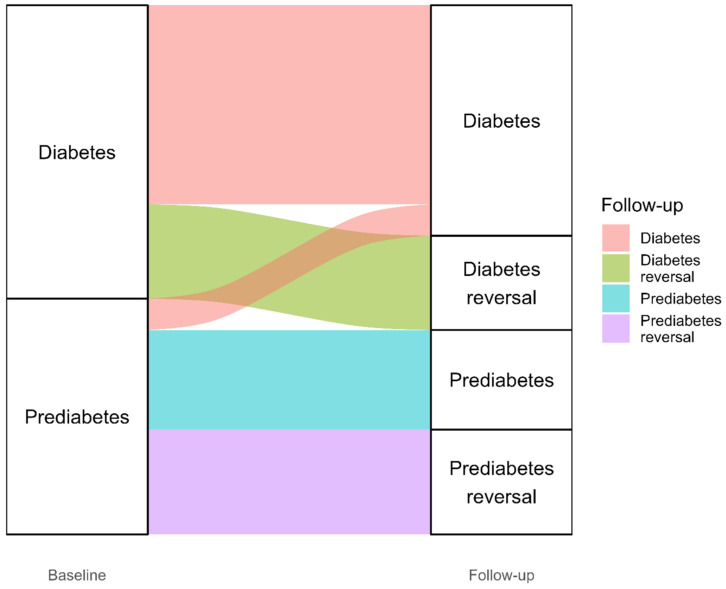
Alluvial chart showing the flow of participants between baseline and final follow-up measurement.

At baseline, 53.3% of patients had T2D while 42.9% had PD. At follow-up, these proportions reduced to 42.9% and 20.9%, respectively. Overall, 32.1% of patients with T2D at baseline achieved reversal, defined as a follow-up HbA1c < 50 mmol/mol, while 44.4% of those with PD at baseline achieved normoglycaemia at final measurement. These outcomes are visually depicted in an alluvial chart (Figure 2), which illustrates the transitions in diabetes status from baseline to the final follow-up.

### 3.3. Factors Associated with HbA1c Change

There was considerable variation in the median number of GP visits between Practices 1 and 3, with Practice 1 having a median of 15 visits (IQR: 8 to 30) and Practice 3 having a median of 2 visits (IQR: 2 to 3) reflecting longer follow-up periods at Practice 1. While 78% of patients from Practice 1 reported engaging with supporting initiatives (such as health coaching, cooking classes or online meetings), only 36% reported engaging at Practice 3.

To investigate factors associated with changes in HbA1c levels (Aim 2), linear mixed-effect models were employed, with results summarised in Table 3. Model 1 included data from all three practices (n = 103. Note: three participant values were missing), while Models 2 (n = 75, Practice 1 and Practice 2) and 3 (n = 51, Practice 1 and Practice 3) were restricted by data availability for specific variables. Across all models, baseline HbA1c was a significant predictor of HbA1c reduction (*p* < 0.001), with higher baseline levels associated with greater decreases (Model 1: coefficient = −0.37, 95% CI: −0.51 to −0.24; Model 2: coefficient = −0.32, 95% CI: −0.51 to −0.24; Model 3: coefficient = −0.4, 95% CI: −0.68 to −0.21).

In Model 1, ethnicity was significantly associated with HbA1c change, with patients of ‘Other’ ethnicities (non-European, non-Māori/Pacific) showing a greater reduction compared to Europeans (coefficient = −12.54, 95% CI: −20.99 to −4.10, *p* = 0.004). Māori or Pacific ethnicity showed a trend toward greater HbA1c reduction (coefficient = −5.62, 95% CI: −13.27 to 2.02, *p* = 0.148). Age and follow-up duration were not significant predictors in Model 1.

**Figure 3:**
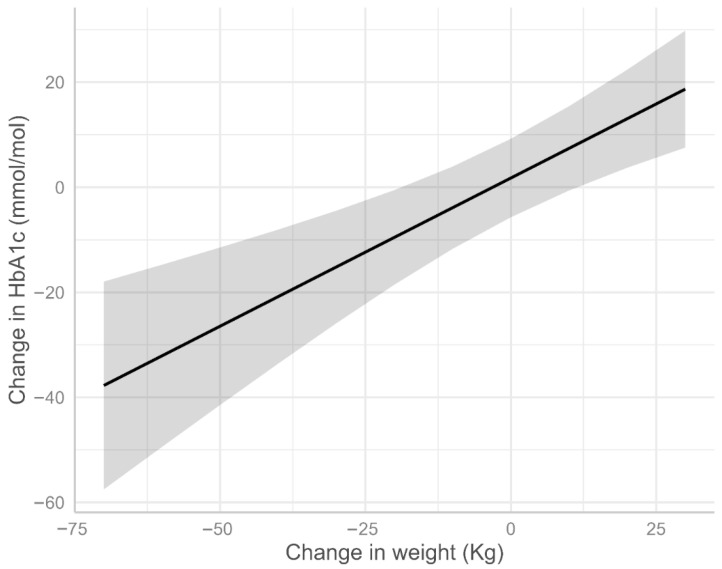
Model-estimated means of HbA1c change by change in weight (based on Model 2 above). The shaded ribbon represents the 95% confidence interval.

Model 2 (Practices 1 and 2), identified a significant association between weight loss and HbA1c reduction (coefficient = 0.56, 95% CI: 0.30 to 0.83, *p* < 0.001), indicating that greater weight loss was associated with larger HbA1c decreases (Figure 3). Follow-up duration showed a small but statistically significant positive coefficient (coefficient = 0.01, 95% CI: −0.01 to 0.01, *p* = 0.011), suggesting a possible trend toward higher HbA1c with longer follow-up. Ethnicity effects were attenuated in Model 2.

In Model 3, which included engagement and frequency of GP visits (Practice 1 and 3), neither engagement (*p* = 0.536) nor the number of GP visits (*p* = 0.698) were significant predictors of HbA1c change. Similarly, the frequency of visits in the initial period showed no significant association (*p* = 0.113). Māori or Pacific ethnicity showed a larger, though non-significant, HbA1c reduction compared to Europeans (coefficient = −26.00, 95% CI: −62.87 to 10.87, *p* = 0.162).

### 3.4. Cardiometabolic Variables

**Figure 4:**
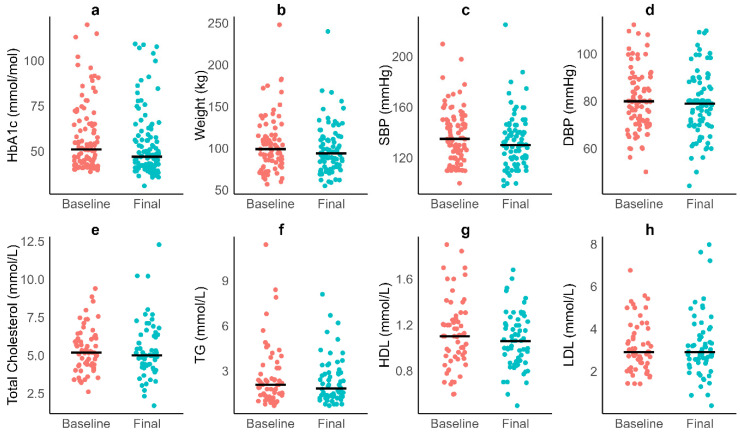
Baseline and final follow-up measurements for (**a**) HbA1c, (**b**) weight, (**c**) systolic blood pressure, (**d**) diastolic blood pressure, (**e**) total cholesterol, (**f**) triglycerides, (**g**) HDL and (**h**) LDL. Black horizontal lines show group medians.

Cardiometabolic variables were assessed between baseline and the final follow-up to evaluate changes associated with the holistic, carbohydrate-reduction model of care (Aim 3). Results are summarised in Table 4, with visual representations in Figure 4.

Weight: Across the cohort (Practices 1 and 2), median weight decreased significantly from 99 kg (IQR: 83 to 115 kg) at baseline to 94 kg (IQR: 78 to 113 kg) at follow-up, with a median change of −3 kg (IQR: −8 to 0 kg). This reduction was statistically significant (Wilcoxon signed-rank test, z = 2276, *p* < 0.001, r = 0.55, large effect), as depicted in Figure 4b.

Blood Pressure: Systolic blood pressure (SBP) showed a median reduction from 135 mmHg (IQR: 120 to 150 mmHg) to 130 mmHg (IQR: 120 to 140 mmHg), with a median change of −3 mmHg (IQR: −20 to 12 mmHg). This change was not statistically significant (z = 1149, *p* = 0.074, r = 0.20, small effect; Figure 4c). Diastolic blood pressure (DBP) remained stable (Figure 4d).

Lipid Profile: Lipid parameters showed mixed results (Figure 4e–h). Total cholesterol (TC) and triglycerides (TG) showed no significant changes (*p* = 0.943, r = 0.01; *p* = 0.680, r = 0.06). HDL cholesterol decreased significantly from 1.10 mmol/L (IQR: 0.92–1.26 mmol/L) to 1.06 mmol/L (IQR: 0.88–1.22 mmol/L; *p* = 0.029, r = 0.31). LDL cholesterol was unchanged (*p* = 0.640, r = 0.07). TG:HDL decreased slightly from 1.69 to 1.65 but this change was not significant (*p* = 0.947, r = 0.01).

Liver Function: Liver enzymes (Practices 1 and 3) showed variable changes. Alanine aminotransferase (ALT) decreased significantly from 33 U/L (IQR: 22–52 U/L) to 23 U/L (IQR: 18–37 U/L; *p* < 0.001, r = 0.53). Alkaline phosphatase (ALP) and gamma-glutamyl transferase (GGT) changes were not significant (*p* = 0.748, r = 0.04; *p* = 0.870, r = 0.03).

Renal Function: Renal markers showed no significant changes. Creatinine, uric acid, and albumin-to-creatinine ratio also remained stable (*p* > 0.379, r ≤ 0.22).

## 4. Discussion

This study provides real-world, clinic-based evidence from three primary care practices, where multidisciplinary teams support patients in applying a holistic approach to managing their PD and T2D. The results demonstrate meaningful improvements in several health markers, most notably including reductions in HbA1c, and body weight. While 44.4% of patients with PD achieved normal HbA1C levels at follow-up, 32.1% of those with T2D at baseline were able to reverse their condition. To our knowledge, this is the first multi-site audit in New Zealand primary care reporting outcomes of carbohydrate reduction in T2D and PD, and it uniquely includes substantial Māori and Pacific representation. Despite variations in approaches across the practices, all utilised a reduced carbohydrate dietary approach and emphasised patient empowerment through knowledge, support initiatives, and a health coach approach. These consistent results across diverse settings suggest that such strategies can be effectively integrated into routine practice. Together, these findings address the study aims by demonstrating improvements in glycaemia (Aim 1), identifying factors associated with change such as weight loss (Aim 2), and describing changes across additional cardiometabolic outcomes (Aim 3).

The success in reducing HbA1c in patients with PD and T2D across all three practices aligns with existing literature supporting the benefits of a reduced carbohydrate approach [22,27,33,34,35,36,37,39,51]. Randomised controlled trials (RCTs) have consistently demonstrated that reducing carbohydrate intake improves glycaemic control [27,34,35,39] by reducing postprandial glucose spikes and improving insulin sensitivity [31]. The extent of HbA1c reduction is often correlated with the degree of carbohydrate reduction, indicating that the benefits may extend beyond weight loss and may involve independent mechanisms related to insulin regulation [31,32,35]. While very-low-carbohydrate or ketogenic diets often yield larger effects [33,37,59], even moderate reduction (<130 g/day) has been shown to produce clinically meaningful improvements [24,33]. In this study, dietary intake was not collected, so the level of carbohydrate reduction achieved cannot be determined. Future audits may benefit from more systematic adherence tracking (e.g., dietary logs or short validated adherence scales) to improve interpretability and strengthen associations between dietary change and clinical outcomes.

In addition to supporting findings from RCTs, this study adds to evidence of effectiveness under real-world clinical conditions. This distinction is important: RCTs establish efficacy under tightly controlled conditions, whereas observational audits capture effectiveness within the constraints, diversity, and competing pressures of routine care. Our findings align with previous clinical practice audits [20,21,22,51], showing improved HbA1c outcomes with carbohydrate reduction in routine care. Given that many patients had lived with PD or T2D for several years, these improvements are particularly notable and challenge the conventional view of T2D as an inevitably progressive disease. These findings are consistent with a substantial body of evidence showing that, under specific conditions, T2D can enter remission in a subset of patients, as reflected in international consensus definitions of diabetes remission [60]. The data indicate that reversal or sustained improvement is achievable even in established cases when structured dietary change and behavioural support are implemented. However, while encouraging, the long-term prognostic implications of diabetes reversal in patients with pre-existing microvascular complications remain uncertain and warrant longitudinal follow-up. Intervening earlier, at the PD stage, may offer the greatest preventive impact, as PD is increasingly recognised as a high-risk and modifiable state [61,62].

Longer disease duration was associated with higher HbA1c at follow-up, but this pattern should be interpreted cautiously. Given the retrospective design and limited sample, no causal inferences can be drawn. Some individuals may have achieved initial improvement before later relapse, reflecting the natural variability of adherence over time. Anecdotal clinician feedback suggests that adherence often fluctuates, with patients cycling between engagement and lapses before re-committing. This aligns with findings from Unwin et al. [22], where longer time on a low-carbohydrate programme correlated weakly with smaller HbA1c improvements, likely reflecting reduced adherence rather than loss of intervention efficacy. However, this is only one possible explanation, and natural disease progression also represents a plausible and simple interpretation of the observed pattern. Importantly, among patients who achieved HbA1c reductions, those with longer disease duration showed greater improvements. This may suggest that the apparent overall trend may be skewed by a subset of patients, who at the time of the last recorded HbA1C measurement were in a phase of low adherence. Taken together, these mixed patterns highlight the need for caution in interpretation and may indicate that multiple factors, including adherence variability and underlying disease progression, may contribute to differences in outcomes over time.

Weight loss was associated with greater reductions in HbA1c, consistent with established links between weight reduction, insulin sensitivity, and improved metabolic health [33]. Ethnic differences were observed but not consistent across models. Patients of “Other” ethnicities showed greater reductions than Europeans, while Māori and Pacific patients showed a non-significant trend toward greater improvement compared to Europeans. These findings should be interpreted cautiously given small subgroup sizes, but they highlight the importance of testing culturally tailored approaches in larger, more powered studies. Importantly, in Practice 1, which serves a high Māori and low socioeconomic population, multiple patients achieved reversal and, among those with reductions, the mean HbA1c decrease was 10 mmol/mol. This is notable, as reversal is less often observed in populations disproportionately affected by diabetes and socioeconomic disadvantage [63,64].

Beyond glycaemic changes, the significant reduction in alanine aminotransferase (ALT) suggests improved hepatic insulin sensitivity and reduced metabolic stress. Elevated ALT, even within normal limits, is a recognised marker of metabolic-syndrome risk as well as a predictor of cardiovascular and diabetes risk, so this improvement adds to evidence of broader metabolic benefit [65]. No adverse lipid effects were observed. In contrast to previous studies [21,51], a slight but clinically insignificant decrease in HDL was observed. This small 3.6% change is within the normal 3–6% biological and analytical variation (6%) for HDL and may partly reflect seasonal effects [66]. Despite this minor decline, both baseline and follow-up HDL values remained above 1.0 mmol/L, within the desired healthy range. More importantly, the triglyceride-to-HDL ratio, a reliable marker of insulin resistance and atherogenic risk [67,68] remained stable, indicating no increase in cardiovascular risk despite the modest HDL reduction.

The quantitative models did not demonstrate a direct association between the frequency of health-coach contact and HbA1c change. However, qualitative feedback and routine observations suggest that ongoing contact, encouragement, and accountability from health coaches were instrumental in sustaining motivation and engagement [69]. This interpretation is consistent with previous studies showing the benefits of a health coaching approach in managing chronic disease [70,71,72,73,74], particularly obesity and diabetes [53]. Although the intensity of contact was not statistically predictive in this dataset, the broader contribution of health coaches likely extends beyond measurable visit frequency. This highlights an important limitation of quantitative metrics, which may fail to capture relational, motivational, and cultural elements of care not easily reflected in numeric contact counts. Such a model could alleviate the burden on GPs while improving patient engagement, provided communication between professionals remains open and aligned [52,75].

This study has several strengths, including exploring outcomes of a comprehensive model of care that integrates health coaches, digital technology, peer support groups, and community initiatives, providing a holistic understanding of the impact of carbohydrate reduction on diabetes management. The inclusion of diverse patient demographics, particularly Māori and Pacific peoples, offers insights into the effectiveness of the model of care across varied groups and highlights pathways to reducing persistent health disparities. These findings highlight the importance of culturally and systemically aligned models of care in populations facing longstanding inequities in access to effective T2D management. Such diversity and pragmatic design enhance generalisability compared with tightly controlled trials. The ability for clinicians to capture and report real-world outcomes, even amid the time pressure, competing demands, and inevitable unpredictability of primary care, remains a key strength of this work.

However, the study is not without its limitations. These include both routine limitations inherent to clinical audit work in primary care and study-specific limitations reflecting the characteristics of the three participating practices. These can be broadly considered in three categories: methodological, data-related, and contextual. Methodological limitations relate to the retrospective and opportunistic audit design. The absence of a control group prevents attribution of changes solely to the intervention, although this was not the primary aim of the audit. We note that opportunistic sampling may have introduced selection bias, particularly at Practice 3, which is known for its diet and lifestyle focus. This could result in a population more motivated or health-conscious than the general diabetic population. However, patients from Practice 3 comprised less than 10% of the cohort, and after accounting for follow-up period we found no evidence of major differences in outcomes between practices.

Data-related limitations include variable completeness between practices with missing data for some clinical measures, and no measures of engagement at Practice 2; this reduced the analytical power for certain outcomes and also limited our ability to address between-site comparisons. Variations in follow-up duration may also have contributed to between-site differences. For triglycerides, no clear pattern was observed, with substantial variation between individuals. This is somewhat unexpected, as reductions in carbohydrate intake are well established to lower triglyceride levels [76]. It is possible that variability in adherence at the time of the last recorded measurement contributed to the noise in these data. Additionally, we cannot be certain that all triglyceride values were fasting, which would have provided a more consistent assessment.

Another key limitation is that detailed dietary intake was not collected, meaning the actual degree of carbohydrate reduction achieved cannot be quantified. This restricts our ability to assess adherence or explore dose–response relationships. Nonetheless, all three practices delivered a consistent whole-food, lower-carbohydrate model of care supported by health coaching, and the outcomes therefore reflect the real-world effectiveness of this approach as implemented. Further, physical activity advice and participation were not systematically recorded across practices, so we were unable to assess or adjust for the contribution of exercise to the observed metabolic changes. Other lifestyle domains commonly addressed within a health-coach–led, holistic model of care, such as sleep, stress management, and smoking, were likewise not routinely captured. These unmeasured factors may have contributed to improvements in some patients and represent important potential confounders for future prospective work to address.

Contextual and practice-related limitations reflect the realities of primary care. Patients at Practice 1 generally had a longer follow-up period than those at Practice 3, due to Practice 3 being newly established, which may have influenced outcome differences. Moreover, routine workflows and competing clinical demands naturally constrained the extent and consistency of data capture across clinics.

Overall, many of these limitations such as the retrospective design, variable follow-up, missing data, and non-standardised measures reflect routine constraints of primary-care audit work, whereas others, such as differences in practice maturity, programme structure, and the specific patient populations served by each clinic, are unique to this study’s three participating sites. They should therefore be understood in context rather than viewed as methodological flaws.

We suggest that our findings highlight the importance of conducting clinical audits and encourage more practitioners to undertake such work in the future. While RCTs remain the gold standard for establishing intervention efficacy, real-world evidence provides a necessary complementary perspective, particularly for understanding the effectiveness of implementation under everyday clinical constraints and for informing service design. Unlike trials, which are often conducted in highly controlled environments with strict participant criteria, real-world data reflects the complexities of everyday clinical practice, including diverse patient populations, challenges of time and funding constraints, and varying adherence levels. Such data offers valuable insights into how integrating lifestyle change performs in broader, less controlled settings, where factors such as comorbidities, context, and access to care can significantly impact outcomes. Given that without intervention, HbA1c levels tend to worsen with increasing T2D duration [77] these findings further highlight the potential effectiveness of the studied approach in a real-world setting. By successfully reducing HbA1c in a population where increases are typically expected, this study provides compelling evidence for the integration of such interventions in everyday clinical practice. As previously mentioned, substantial and robust evidence supports therapeutic carbohydrate reduction, which has consequently been incorporated into diabetes management guidelines worldwide [42,43,44]. Future research should focus on prospective evaluations in routine care, particularly within Māori and Pacific communities, alongside assessments of implementation fidelity, health-coach integration, and the contribution of digital and peer-support tools. Incorporating more systematic adherence tracking, such as structured logs would also strengthen future analyses Such work will help identify how best to optimise and scale these approaches within different primary care contexts.

## 5. Conclusions

This multi-site audit suggests that integrating whole food, carbohydrate-reduction strategies within holistic, health-coach-supported, and community-engaged models of care may support meaningful metabolic improvements for patients with PD and T2D in real-world primary care. While the observational design and variability across practices limit causal interpretation, the consistency of outcomes across varied settings and populations supports the feasibility and potential impact of this approach in real-world diabetes management.

## Figures and Tables

**Table 1:** Metabolic markers measured at each practice.

Measure	Practice 1	Practice 2	Practice 3
HbA1c	✓	✓	✓
Weigh	✓	✓	
Blood pressure	✓	✓	
Lipids	✓	✓	✓
Renal function	✓		✓
Liver function	✓		✓

**Table 2:** Demographic data for each practice. Note European includes NZ European and ‘other’ European.

	Practice 1	Practice 2	Practice 3	Total
Patients (n, %)	46 (43%)	41 (39%)	19 (18%)	106
Female (%)	61	40	63	53
Male (%)	39	60	37	47
Ethnicity	52% Māori, 41% Pacific Island	89% European	73% European	-
Mean Age (years ± SD)	46.4 ± 11.3	70 ± 16.0	54.5 ± 10.5	54.0 ± 17.0
Median Follow-up Duration (months, IQR)	38 (13–50)	19 (8–23)	5 (3–9)	19 (6–32)

**Table 3:** Factors associated with change in HbA1c. Bolded values denote significant test results (*p* < 0.05).

Variable	Coefficient	95% CI	*p* Value
Model 1 (n = 103, all practices)			
Age (years)	−0.14	−0.34, 0.06	0.154
Follow-up duration (days)	<0.01	−0.01, 0.01	0.095
Baseline HbA1c (mmol/mol)	−0.37	−0.51, −0.24	**<0.001**
Ethnicity			
European	—	—	
Māori or Pacific	−5.62	−13.27, 2.02	0.148
Other *	−12.54	−20.99, −4.10	**0.004**
Model 2 (n = 75, Practice 1 and 2)			
Age (years)	−0.08	−0.33, 0.14	0.498
Follow-up duration (days)	0.01	−0.01, 0.01	0.011
Baseline HbA1c (mmol/mol)	−0.32	−0.51, −0.24	**<0.001**
Ethnicity			
European	—	—	
Māori or Pacific	−0.88	−10.17, 8.42	0.851
Other *	−14.39	−28.86, 0.08	0.051
Change in weight (Kg)	0.56	0.30, 0.83	**<0.001**
Model 3 (n = 51, Practice 1 and 3)			
Age (years)	−0.23	−0.66, 0.20	0.467
Follow-up duration (days)	<0.01	−0.01, 0.01	0.142
Baseline HbA1c (mmol/mol)	−0.44	−0.68, −0.21	**<0.001**
Ethnicity			
European	—	—	
Māori or Pacific	−26.00	−62.87, 10.87	0.162
Other *	−36.18	−76.76, 4.40	0.079
Engagement			
Yes	—	—	
No	3.42	−7.64, 14.48	0.536
Number of doctor’s visits (n)	0.07	−0.31, 0.46	0.698
Frequency initial period (times/month)	−0.94	−2.11, 0.23	0.113

* ‘Other’ ethnicity includes Indian, Sri Lankan, Chinese, Southeast Asian, Asian not further defined, other Asian, African, Latin American, Middle Eastern.

**Table 4:** Statistical analysis of cardiometabolic variables measured at baseline and at the end of the evaluation period. Data are presented as median (25th, 75th percentile). Change scores were calculated for each participant (final—baseline), and the median of these values is reported. *p* values from Wilcoxon signed-rank test. ALP: Alkaline Phosphatase; GGT: Gamma-Glutamyl Transferase; ALT: Alanine Aminotransferase. Bolded values denote significant test results (*p* < 0.05).

Variable	Baseline	Last Follow-Up	Change	Matched Pairs	r	*p* Value
Weight (kg)	99 (83, 115)	94 (78, 113)	−3 (−8, 0)	77	0.55	**<0.001**
HbA1c (mmol/mol)	51 (42, 65)	47 (41, 49)	−3 (−7, 3)	103	0.28	**0.005**
Systolic blood pressure (mmHg)	135 (120, 150)	130 (120, 140)	−3 (−20, 12)	83	0.20	0.074
Diastolic blood pressure (mmHg)	80 (70, 88)	79 (70, 88)	0 (−4, 0)	83	0.17	0.148
Total Cholesterol (mmol/L)	5.20 (4.40, 6.10)	5.00 (4.20, 6.40)	0.05 (−0.63, 0.43)	49	0.01	0.943
Triglycerides (mmol/L)	2.09 (1.38, 3.20)	1.85 (1.30, 2.90)	0.01 (−0.90, 0.42)	48	0.06	0.680
HDL (mmol/L)	1.10 (0.92, 1.26)	1.06 (0.88, 1.22)	−0.11 (−0.21, 0.07)	49	0.31	**0.029**
LDL (mmol/L)	2.90 (2.30, 3.90)	2.90 (2.40, 3.75)	0.00 (−0.45, 0.48)	42	0.07	0.640
TG:HDL radio	1.69 (1.06, 3.36)	1.65 (1.13, 3.39)	0.03 (−0.85, 0.89))	48	0.01	0.947
ALP (u/L)	79 (46, 102)	74 (57, 89)	−1 (−16, 11)	46	0.04	0.748
GGT	35 (26, 58)	33 (21, 57)	−4 (−15, 18)	46	0.03	0.870
ALT	33 (22, 52)	23 (18, 37)	−10 (−20, −1)	42	0.53	**<0.001**
Creatinine (µmol/L)	67 (60, 85)	66 (58, 82)	−2 (−7, 7)	42	0.06	0.726
Uric acid (mmol/L)	0.37 (0.33, 0.40)	0.38 (0.34, 0.41)	0.01 (−0.01, 0.03)	16	0.21	0.379
Albumin-to-creatinine ratio	1 (0, 2)	1 (0, 2)	0.02 (−0.01, 0.24)	16	0.22	0.505

## Data Availability

The data that support the findings of this study are available from the corresponding author upon reasonable request.
